# Clinician and Visitor Activity Patterns in an Intensive Care Unit Room: A Study to Examine How Ambient Monitoring Can Inform the Measurement of Delirium Severity and Escalation of Care

**DOI:** 10.3390/jimaging10100253

**Published:** 2024-10-14

**Authors:** Keivan Nalaie, Vitaly Herasevich, Laura M. Heier, Brian W. Pickering, Daniel Diedrich, Heidi Lindroth

**Affiliations:** 1 Division of Nursing Research, Department of Nursing, Mayo Clinic, Rochester, MN 55905, USA; nalaie.keivan@mayo.edu (K.N.); heier.laura@mayo.edu (L.M.H.); 2 Department of Anesthesiology and Perioperative Medicine, Mayo Clinic, Rochester, MN 55905, USA; vitaly@mayo.edu (V.H.); pickering.brian@mayo.edu (B.W.P.); diedrich.daniel@mayo.edu (D.D.); 3 School of Nursing, Viterbo University, La Crosse, WI 54601, USA; 4 Center for Aging Research, Regenstrief Institute, School of Medicine, Indiana University, Indianapolis, IN 46202, USA; 5 Center for Health Innovation and Implementation Science, School of Medicine, Indiana University, Indianapolis, IN 46202, USA

**Keywords:** people counting, intensive care, object detection, computer vision, health care, hospital, delirium

## Abstract

The early detection of the acute deterioration of escalating illness severity is crucial for effective patient management and can significantly impact patient outcomes. Ambient sensing technology, such as computer vision, may provide real-time information that could impact early recognition and response. This study aimed to develop a computer vision model to quantify the number and type (clinician vs. visitor) of people in an intensive care unit (ICU) room, study the trajectory of their movement, and preliminarily explore its relationship with delirium as a marker of illness severity. To quantify the number of people present, we implemented a counting-by-detection supervised strategy using images from ICU rooms. This was accomplished through developing three methods: single-frame, multi-frame, and tracking-to-count. We then explored how the type of person and distribution in the room corresponded to the presence of delirium. Our designed pipeline was tested with a different set of detection models. We report model performance statistics and preliminary insights into the relationship between the number and type of persons in the ICU room and delirium. We evaluated our method and compared it with other approaches, including density estimation, counting by detection, regression methods, and their adaptability to ICU environments.

## 1. Introduction

Each year, upwards of 5.7 million patients are admitted to intensive care units (ICUs) across the United States [1]. Proactively anticipating the resource needs of critically ill patients and factors is important for optimizing resource utilization and patient management. Several health information technologies have been developed leveraging electronic health record (EHR); however, these are inherently impacted by delayed documentation or the structure of the EHR data. Ambient sensor technology, like computer vision, can provide real-time information to administrators and clinicians on the resource needs of a patient, while also providing visual cues that illness severity is increasing, or that an acute deterioration is eminent. One such condition that is associated with increasing illness severity is delirium, an acute brain dysfunction [2]. Approximately 50% of patients will experience delirium in the ICU, leading to a heightened risk of mortality and future dementia [2,3,4,5]. A previous study reported that patients with delirium have more caregiver activity between the hours of 8:00 p.m. and 12:00 a.m. [6]. Detecting and quantifying this clinician activity could inform unit staffing needs, including 1:1 patient care assignments. Furthermore, delirium is often a warning sign indicating an acute deterioration or other physiological need [7,8]. Artificial Intelligence (AI) algorithms that use ambient sensing data to forecast the probability of such outcomes may improve patient management and ICU care efficiencies.

One potential data source for an AI algorithm using ambient sensor data to forecast patient resource needs is the number and type of people in an ICU room at any given time. This data-source-using a computer vision task is named “visual people counting”, and it has gained significant attention due to its diverse applications across various industries. These include public transportation [9], smart cities [10], and health care facilities, where it plays a crucial role in managing patient flow [11] and controlling infections [12,13]. Generally, techniques for people counting fall into two main categories: supervised and unsupervised methods. Unsupervised methods, as detailed in some studies [14,15], typically involve clustering to perform counting. Supervised people counting can be further categorized into counting by detection [16,17,18], counting by regression [19,20], and counting by density estimation [21]. Publically available object detection datasets typically comprise a vast number of images, where objects of interest are marked with bounding boxes. For instance, the Pascal Visual Object Classes (VOC) dataset [22] contains 11,530 images with 27,450 annotated objects, including general categories such as persons, vehicles, and animals. More recently, the HOID dataset [23] was introduced, featuring 4417 images annotated with 56 object categories specific to hospital settings, covering areas like intensive care units, operating rooms, and hospital waiting areas. These datasets, while comprehensive, do not capture the hour-by-hour changes within an ICU room that contains a patient, leading to difficulty in constructing an AI algorithm.

We aimed to overcome this challenge using a sub-dataset from an ongoing computer vision study that prospectively enrolls ICU patients and captures the image data of their environment in real time. Our goal was to create a computer vision-based system that accurately counts the number of people in an ICU room, such as visitors, clinicians, and patients. We use counting-by-detection, which leverages a relatively simple network architecture that enables us to perform in real time. This feature is particularly beneficial in the dynamic and complex environment of the ICU. Due to the dynamic nature of the scene, where objects may be overlapped or occluded, people based solely on a single image poses challenges. Therefore, we developed single-frame, multi-frame, and counting-by-tracking models. We then preliminary explored the relationship between the number of people detected by the model and the presence of delirium. We contribute the following to the field of computer vision: (1) single-frame, multi-frame, and tracking-to-count approaches for people counting, (2) an evaluation of these different approaches with several metrics, (3) and the preliminary exploration of the relationship between the number of people detected and the presence of delirium.

### 1.1. Methods and Materials

As part of an ongoing prospective study, we analyzed image data from fifty-two ICU patients with 4× daily delirium severity assessments. Full informed consent was obtained from the patient or the identified legally authorized representative following approval from the Institutional Review Board (IRB, 22-003098, 20-006366). To protect the privacy of the individuals shown in the images, no identifiable information was used in the analysis (i.e., no facial recognition, images were blurred). The study team posted several 8 × 10-inch bright yellow posters inside and outside of the room so all individuals going into the room were aware of the continuous video recordings.

### 1.2. Image Collection and Dataset Preparation

Following informed consent, two GoPro10 cameras were set up in the patient’s ICU room. The first camera was placed at the end of the patient’s bed. The second camera was placed adjacent to the patient’s bed to capture more contextual details. These cameras were positioned 3–5 m away from patients, with a resolution of 5568 × 4176 pixels and a frame rate of one frame per second. Images were collected continuously for 72 h, or until the patient discharged or died, in a variety of different ICU rooms. The variation in ICU rooms provided diversity in setting and patient type. Following image capture, the data were prepared in a three-step process, as outlined below.

**Object category selection.** We chose to identify three categories of individuals who frequently enter the ICU room: patients, clinicians, and visitors. The patient category is crucial for verifying the presence of the patient in the room, a key factor in assessing the efficacy of our ambient monitoring system. Thus, including the patient category helps ensure the accuracy of our model regarding patient presence. Clinicians are included because their actions have a direct impact on patient outcomes. Visitors, defined as individuals other than clinicians (i.e., not wearing scrubs) could represent family members, friends, or patient services such as spiritual care, are included due to their role in influencing patients’ emotional and psychological well-being, which can impact recovery.

**Image cleaning**. To ensure that our object detection model was trained with high-quality images, we excluded any that were blurry, of low resolution, partially occluded, or in complete darkness. This image cleaning process involved a thorough review of the collected images to remove those that did not meet our criteria. After eliminating 244 images due to these issues, 2130 images remained, which were deemed suitable for further analysis.

**Figure 1:**
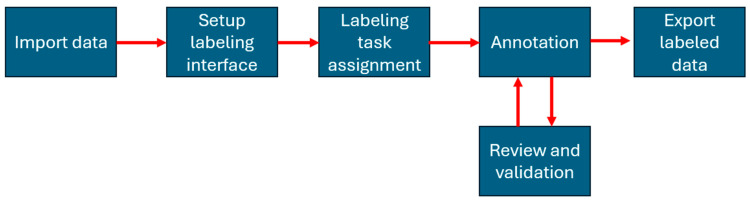
Image annotation process diagram. We begin by uploading images from local storage into Label Studio. After configuring the labeling environment to meet the project’s requirements, we assign a team member as the annotator. This annotator labels the images by drawing bounding boxes around the subjects of interest. To maintain consistency and ensure quality, the same person reviews and validates the annotations. Finally, we export the annotated data in formats like JSON and COCO, which are then used for model training.

**Image annotation**. We utilized LabelStudio [24] for image annotation as it supports multi-class object identification within a single frame. In the annotation software, we annotated collected images by identifying objects using bounding boxes. To maintain consistency in the annotations, a single individual managed the entire dataset. Furthermore, to ensure the quality of the annotations, this person also performed a comprehensive assessment after the initial annotation process was complete. Figure 1 presents the image annotation process.

In total, the dataset comprises 2130 images. These were annotated to identify patients, clinicians, and visitors for a total of 5674 annotated objects (2062 patients, 2048 clinicians, 1564 visitors).

### 1.3. Patient and Clinical Data Collection

Demographic and clinical characteristics of enrolled patients were extracted from the electronic health record (EHR). Delirium and delirium severity were assessed using the gold standard in the ICU, the Confusion Assessment Method for the ICU (CAM-ICU) and the CAM-ICU-7 (scored directly from the CAM-ICU on a 0–7 scale). Higher scores indicate more severe delirium. These validated assessments were administered 4× daily by trained study team members.

### 1.4. Data Structure and Representation

**Figure 2:**
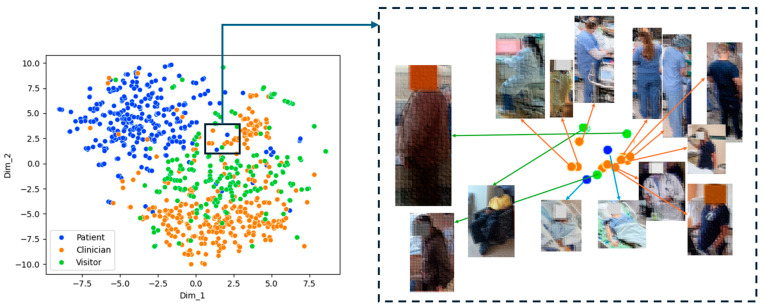
t-SNE results on the validation/test set, featuring 5674 annotated objects, 2062 patients, 2048 clinicians, and 1564 visitors. Due to privacy considerations, images of individuals are de-identified and blurred.

To clarify and structure our dataset, we employed the t-SNE algorithm to reduce high-dimensional data points to a two-dimensional format and then to cluster and visualize that data, as shown in Figure 2. Furthermore, we extract visual features for each object, where each bounding box within an image is processed through a network [25] trained to identify and quantify these features, resulting in a high-dimensional data representation at 1000 dimensions. The t-SNE algorithm effectively minimizes the divergence between two distributions: the original high-dimensional proximity of data points and their new two-dimensional arrangement. This reduction strategy generates a visual map where points representing similar objects are placed in proximity, while points representing dissimilar objects are spaced further apart. Because t-SNE preserves local neighborhood structures during dimensionality reduction, it is more effective than linear methods such as PCA in producing interpretable low-dimensional representations, especially for visualization purposes.

As illustrated in Figure 2, three clusters of individuals are observed. Patients predominantly cluster in the upper section of the plot, indicating that their visual features are distinct from other categories. The categories of clinicians and visitors exhibit some degree of clustering with notable overlap. This overlap suggests that the visual features used to annotate these two groups might share similarities, potentially due to overlapping elements in their appearances or physical characteristics in the ICU room. This insight is crucial for refining our algorithms and improving the accuracy of class distinctions within the dataset.

**Figure 3:**
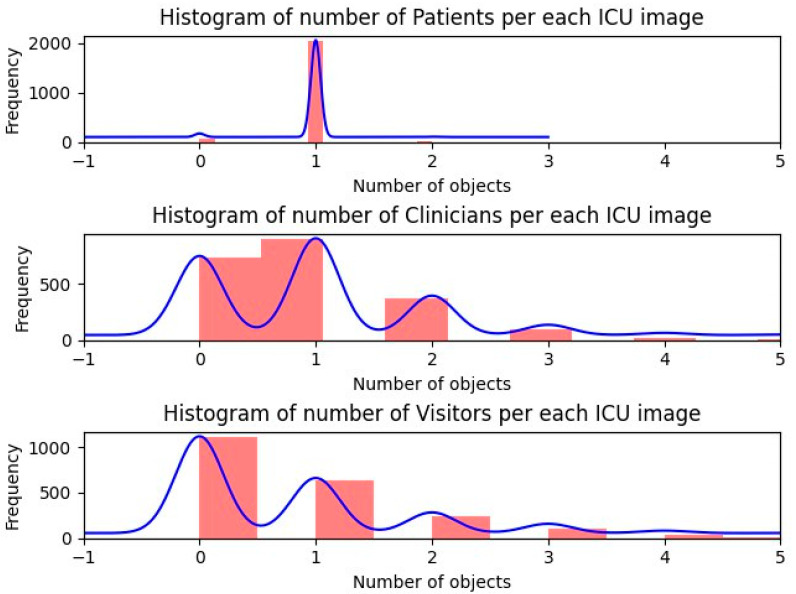
Histogram plots of object categories. The charts illustrate the frequency distribution of detected objects in three categories: patients, visitors, and clinicians. The patient category exhibits minimal variability, indicating uniformity across measurements. In contrast, the visitor and clinician categories demonstrate greater variability with multiple peaks, reflecting diverse roles and interactions within the ICU setting.

Figure 3 offers a detailed analysis of the histogram distributions for each object category within ICU rooms. It illustrates that most images captured feature only one patient per room, indicating a common scenario in these settings. In contrast, the distributions for visitors and clinicians show differing patterns. The clinician category typically concentrates around the presence of zero or one clinician per room, suggesting that it is common to have at most one clinician present at any given time. On the other hand, the distribution for visitors is more varied and extends further along the x-axis. This shows more variability in how many visitors might be present in different scenarios within the ICU.

## 2. Model Development

**Figure 4:**
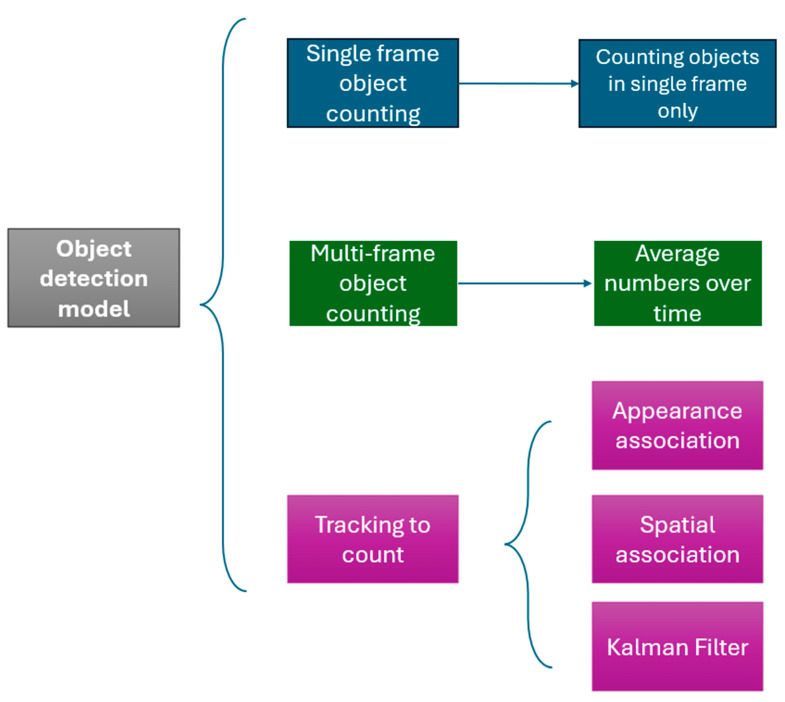
Methodology overview: schematic summary.

To achieve a people counting pipeline, we used an object detection framework [26] to gauge room occupancy and used three distinct methodologies: single-frame object counting, multi-frame object counting, and object tracking. A schematic summary of our methodology is presented in Figure 4. We split the dataset into training (80%) and validation (20%) sets.

**Single-frame object counting**. Our single-frame object counting model, employed an object detection pipeline to detect and localize objects of different categories within each frame. The result is a detailed output that includes the coordinates and the category of each detected object. We then use those detection results to count the number of objects per frame based on their specific categories. Moreover,

(1) $$Out_{i}=Count\left[Det\left(Image_{i}\right)\right]\in \;N^{C}$$

where $Image_{i}$ is the i-th input frame, function Det stands for the object detection model, and function Count counts the number of detected objects in each of the C categories. In our study, we defined C=3 to represent the three object categories: patient, visitor, and clinician.

**Multi-frame object counting.** Next, we improved our people counting technique by expanding it to encompass multiple frames. Specifically, for the i-th input frame, we performed counting for the F/2 frames preceding and succeeding frame i. This process included accumulating counts from these frames and calculating the average number of objects detected per frame. By applying counting across multiple frames, this technique helped to smooth out any noise and anomalies that may impact the detection model’s performance. For instance, if an object is obscured or hidden behind other objects in the scene, counting over several frames helps to overcome this challenge, leading to a more consistent and precise outcome. The mathematical formulation of this approach is as follows:

(2) $$Out_{i}=\frac{1}{F}\sum_{j=i-F/2}^{i+F/2}Count\left[Det\left(Image_{j}\right)\right]\in \;N^{C}$$

where F represents the size of the window frame defined around the *i*-th input frame.

**Tracking to count**. The ability to count objects in an ICU room relies heavily on the detection model’s capability to precisely identify and categorize objects. As mentioned earlier, extending the time frame to include more frames and averaging the results can improve the accuracy of the model, especially in cases of missing information. However, patients, visitors, and clinicians often exhibit similar physical appearances, which can challenge the model’s ability to accurately classify these distinct categories. For example, a detection model might initially fail to accurately detect a clinician due to the distance from the camera or because of obstructions. As the clinician moves closer or the obstruction is reduced, the model’s ability to correctly identify the category may improve. To address these challenges, we employed a multi-object tracking solution that tracks identified objects over time.

In multi-object tracking, individuals are tracked across a series of F frames. An object tracker links detected objects from one frame to the next, using spatial coordinates and visual characteristics to maintain accurate and continuous monitoring. These links create a trajectory for each individual over multiple frames. In environments with dynamic challenges such as low visibility or object occlusion, the method of counting trajectories is more advantageous than relying solely on detections. Below, we explain the application of tracking to counting objects in the ICU setting.

### 2.1. Tracking-to-Count Model

People counting in the ICU can be formulated as a multi-object tracking problem: given F input frames $X=\left\{{Image}_{1},{Image}_{2},\ldots ,{Image}_{F}\right\}$, where $x_{i}\in R^{3\times H\times W}$ (RGB image), we first aim to extract $B_{i}=\{b_{i,1},b_{i,2},\ldots ,b_{i,Mi}\;\}$ as a set of detected bounding boxes within frame i, where $b_{i,j}=\left\{rect_{i,j},\phi_{i,j}\right\}$, $rect_{i,j}$ stands for the j-th detected bounding box (center of coordinates, width and height) from the object detector (e.g., Faster RCNN), and $\phi_{i,j}$ is the extracted re-identification (re-ID) feature, serving to capture distinct appearance characteristics within each bounding box $b_{i,j}$. This enables us to consistently identify and differentiate individuals across frames and varying conditions. Then, object tracking aims to associate bounding boxes to construct sets of trajectories $T_{i}=\{B_{i},id_{i}\}$, where $B_{i}$ is a set of bounding boxes, and $id_{i}$ stands for trajectory ID within frame i.

**Figure 5:**
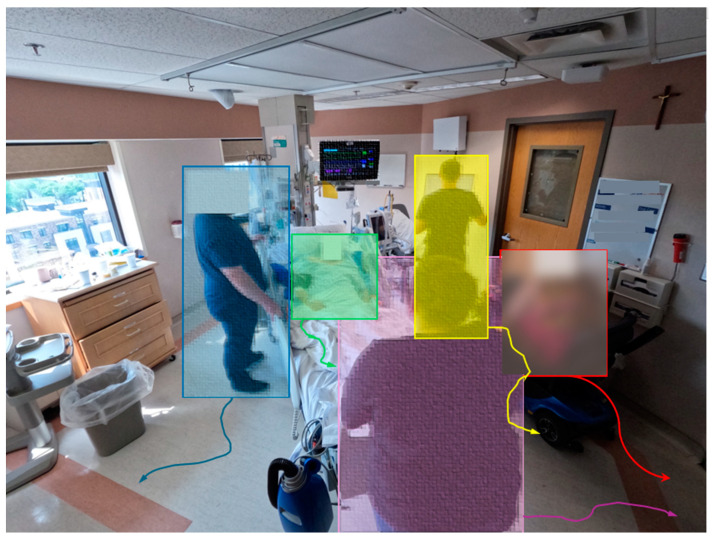
Visualization of constructed trajectories up to frame *i*. The tracking mechanism associates objects up to frame *i* to construct a set of objects, each characterized by a series of appearance features and spatial coordinates. Due to privacy considerations, images of individuals are de-identified and blurred.

**Figure 6:**
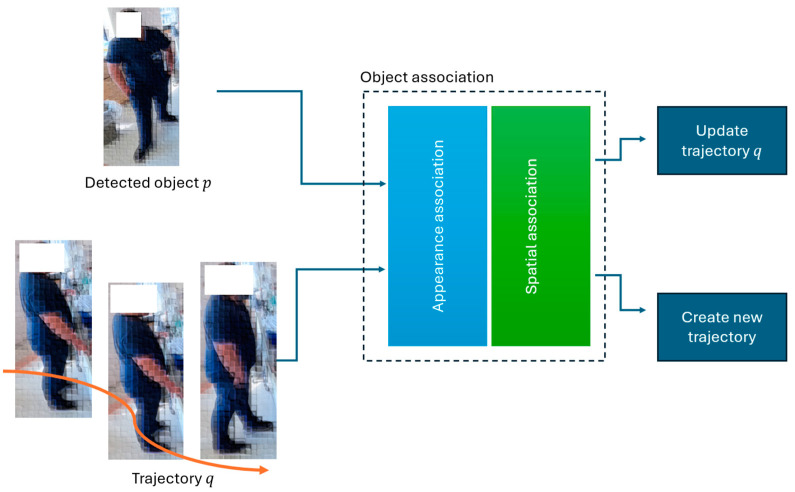
Multi object tracking mechanism. Our tracking pipeline operates across a sequence of multiple frames. In each frame, it seeks to associate detected objects (e.g., object *p*) with all existing trajectories (e.g., trajectory *q*) that have been constructed up to that frame. If a detected object is associated with one of the existing trajectories, that trajectory is extended; otherwise, a new trajectory is initiated. Due to privacy considerations, images of individuals are de-identified and blurred.

We associated detected objects from one frame to the next by calculating an affinity matrix using cosine similarities between the features of detected objects. This matrix helps to determine the likelihood that two objects are the same entity. Figure 5 presents example of visualization of constructed trajectories. Similarly [27], we evaluate the similarity between each trajectory and each detected object through bipartite matching, with the goal of maximizing the similarity scores on the edges connecting current frame detections to the existing trajectories. For tracking people within an ICU, our method leverages both the appearance (how objects look) and spatial similarities (where objects are located) of detected objects (Figure 6) across F frames to map each detected object to a unique trajectory.

The initial step involves matching the re-ID (appearance) features of newly detected objects with those of existing trajectories, thereby creating a robust appearance affinity matrix that facilitates accurate tracking. In the first step, for each detected object $b_{i,j}$ in a set of detected objects $B_{i}$ at frame i, we analyze the visual content within the bounding box by running it through a re-identification (re-ID) network [25]. If the detection does not match any existing trajectories, we proceed to a secondary verification step. In the second step, the model assesses whether the currently detected object significantly overlaps with any existing trajectories using the Intersection over Union (IoU) metric. If a match is found, the corresponding trajectory is updated to incorporate the new detection; if not, a new trajectory is started. In addition, the model employs a Kalman Filter [28] to update the coordinates of the detected trajectories. This Kalman Filter helps predict the future positions of each trajectory, thereby improving the model’s capacity to effectively track the activity of individuals in the ICU room.

Each constructed trajectory represents an individual in the room. Additionally, we classify the object category of each trajectory using the category probability distribution from each box $b_{k,j}$ within trajectory $Trj_{k}$. The probability function for an object category related to box $b_{k,j}$ is denoted as $P_{k,j}$ and $j\in \left[1,M_{k}\right]$, where $M_{k}$ is the length of the k-th trajectory. Essentially, $P_{k,j}$ is a value derived from the detection backbone of the model, which quantifies the likelihood that the j-th detected bounding box of trajectory $Trj_{k}$ belongs to a certain category. We then determine the object category for trajectory $Trj_{k}$ as follows:

(3) $$Category\left(Trj_{k}\right)=arg\;\underset{c}{\max}\sum_{j=1}^{M_{k}}P_{k,j}\;\;,P_{k,j}\in \;R^{C}$$

Thus, the category of each trajectory is determined by the likelihood associated with its bounding boxes throughout its duration.

### 2.2. Model Performance Evaluation

**Object detection.** We measured the mean average precision (mAP) to evaluate the performance of the object detection models. The precise localization of each object was evaluated using the Intersection over Union (IoU) metric (>0.5 valid detection).

Each bounding box prediction is associated with a confidence score, which influences the precision and recall calculations. Generally, a higher confidence score might improve precision but could potentially lower recall. The average precision (AP) for each category is defined as the average of precision values calculated at different recall levels (r), ranging from 0 to 1 in increments of 0.01:

(4) $$\mathrm{AP}=\frac{1}{101}\sum_{r\in \left\{0,0.01,\ldots ,1\right\}\;}P_{r}$$

The overall AP score combines the precision and recall scores into a single metric. As the IoU threshold increases, typically, precision increases, but the AP score decreases due to stricter evaluation criteria. To address variations in IoU thresholds, the mAP is calculated as the mean of AP scores across multiple IoU thresholds, specifically ten thresholds ranging from 0.5 to 0.95:

(5) $$\mathrm{AP}@[0.5:0.95](\mathrm{mAP})=\frac{1}{10}\sum_{IoU\in \left\{0.5,..,0.95\right\}\;}AP_{IoU}$$

The mAP is averaged across various IoU threshold values, providing a comprehensive evaluation of a model’s detection performance across varying levels of detection difficulty.

**People counting.** We evaluated the effectiveness of the people counting model using three key metrics: accuracy and Mean Absolute Error (MAE), Mean Relative Error (MRE), and Mean Square Error (MSE). The accuracy metric measures how precisely the model counted the number of people in each predefined category (patient, clinician, visitor) compared to the actual numbers (ground truth). The equations for the MAE, MRE, and MSE are as follows:

(6) $$\mathrm{MAE}=\frac{1}{n}\sum_{i=1}^{n}\left|y_{i}-\hat{y_{i}}\right|,\;\;MRE=\frac{1}{n}\sum_{i=1}^{n}\frac{\left|y_{i}-\hat{y_{i}}\right|}{y_{i}},\;\;MSE=\frac{1}{n}\sum_{i=1}^{n}{\left(y_{i}-\hat{y_{i}}\right)}^{2}\;$$

where $y_{i}$ is the actual value, $\hat{y_{i}}$ is the predicted value, and n is the number of points.

**Delirium.** As these data were part of an ongoing prospective study, we lacked the statistical power to formally test the relationships between the number of clinicians and/or visitors in the room and the level of delirium severity. Instead, we descriptively examined the data between the presence/absence of delirium (CAM-ICU positive/negative) and subsyndromal delirium/mild-to-severe delirium (CAM-ICU-7 score of 1-2/3-7).

## 3. Results

A total of fifty-two patients were included in the model development with a mean age of 66.90 (SD 9.47), 37% female, and mostly Caucasian.

### 3.1. Implementation

Our network was trained on a workstation equipped with an Intel Xeon Silver 4114 CPU, 64 GB of RAM, and an 2xNVIDIA GTX 1080 Ti GPU, operating under Ubuntu 22.04.3. The network’s initial weights were initialized from models previously trained on the COCO dataset [29]. We implemented our model using Python 3.8 and Detectron2 [26] an open source object detection platform that features leading algorithms such Retinanet/Faster R-CNN with different backbone combinations: FPN and DC5. Retinanet and Faster R-CNN are both widely used object detection models, but they differ in their design and approach. RetinaNet is a single-stage detector that utilizes focal loss to address class imbalance, making it effective for detecting small objects in large datasets. Faster R-CNN is a two-stage detector that first generates region proposals and then refines them to classify objects. For the Feature Pyramid Network (FPN), we utilized a Resnet/Retinanet and FPN backbone that includes standard convolution layers and fully connected heads for predicting masks and boxes. For the DC5 configuration, which incorporates deformable convolution, we employed a Resnet conv5 backbone that applies dilation in the conv5 layer, along with conventional convolution and fully connected heads for mask and box prediction. We used transfer learning by leveraging pretrained weights from the COCO object detection baseline for both configurations. The training parameters included a learning rate of $2.5\times 10^{-4}$ and a total of 16 K iterations. For training, we used standard data augmentation techniques, provided by the Detectron2 library, which included scale jittering and horizontal flipping. Table 1 presents a summary of the parameter configuration in our pipeline. We allocated 20% of our dataset for testing and used the remaining 80% for training. Given the varying sizes of the model architectures, the input image dimensions, and the available GPU memory, we opted for a batch size of 4 images. We conducted 16 K training iterations across the entire dataset, which resulted in satisfactory testing accuracy. Fewer iterations led to insufficient reduction in training loss, while increasing the number of iterations resulted in overfitting—yielding high accuracy on the training set but lower accuracy on the testing set.

### 3.2. Object Detection Performance in ICU Room

Table 2 compares the performances of five object detection models across different metrics. Faster RCNN with Resnet-FPN features the shortest inference and training times due to its smaller parameter size, achieving an average precision (AP) of 52.37. However, it showed the lowest detection rates across all object categories. In contrast, Faster R-CNN with Resnet50-DCN had longer inference and training times due to a larger parameter size but achieved a slightly higher AP of 53.25. Faster R-CNN with Resnet101-FPN, despite a smaller parameter size, recorded an AP of 54.06 and the highest detection rates for clinicians at 59.91, alongside a balanced performance for patients and visitors. Retinanet with Resnet50-FPN required longer training times and achieved a similar detection performance to the preceding model. Retinanet with Resnet101-FPN, while the most resource-intensive, was also the most accurate model with an AP of 54.60 and notable detection rates of 51.06, 59.30, and 53.53 for patients, clinicians, and visitors, respectively.

It is also important to note that across all models, the clinician category was detected with the highest accuracy, followed by the visitor and patient categories.

### 3.3. People Counting Results in ICU Room

We report the performance of various approaches in people counting in Table 3.

**Single-frame people counting**. This technique employs an object detector to identify various objects across different categories within keyframes, subsequently counting these objects to evaluate their distribution. The success of this approach hinges on the performance of the object detection model. According to Table 3, the Faster R-CNN model using Resnet50-FPN offered the highest accuracy in counting objects from each category, as evidenced by the low MSE, RMSE, and MAE values across different evaluations. Conversely, the Resnet-50 DC5 model recorded the lowest accuracy for clinicians (70.96) and exhibited the highest error rates compared to other models. Additionally, the Retinanet model with a Res-net101-FPN backbone demonstrated the weakest performance, underscoring the variability in these models’ effectiveness in object detection and counting.

**Multi-frame people counting.** As shown in the second row in Table 3, we analyzed the impact of multi-frame processing on people counting accuracy. In our experiments, we processed images at a frame rate of one frame per second. This multi-frame approach involves performing object detection on frames surrounding each keyframe, effectively analyzing a set of F frames (Duration) centered around the keyframe. We then averaged the object category distributions across these F frames. The data from Table 3 clearly show that the accuracy scores are generally higher in the multi-frame scenario compared to the single-frame scenario, as seen when comparing the first row to the second row. For example, when employing multi-frame processing, the accuracy of RetinaNet with ResNet101 improved by 4.98% and 7.92% for the clinician and visitor categories, respectively. A similar trend is observed in the reported error values, where multi-frame processing resulted in reduced regression errors for the clinician and visitor categories.

**Tracking to count**. In the third row of Table 3, we evaluated the impact of object tracking on the accuracy of people counting, particularly in an ICU setting. The comparison of the object tracking results with those of other methods underscores object tracking’s critical role in enhancing people counting, especially with dynamic entities like clinicians and visitors. Both accuracy and error values generally improved with the use of object tracking, a benefit that is most pronounced with moving objects. Interestingly, while single-frame approaches demonstrated lower performance, particularly the RetinaNet models, incorporating object tracking notably enhanced the people counting accuracy. Furthermore, Faster R-CNN models with Resnet101 achieved the highest accuracy in counting clinicians at 77.71%, and RetinaNet models with Resnet-50 showed the best accuracy for counting visitors at 79.17%.

The results from Table 3 demonstrate that multi-frame analysis and tracking methods significantly enhance the accuracy of counting clinicians and visitors as opposed to patients. The frequent movement of clinicians and visitors often resulted in complexities like occlusion and overlapping in the scene, issues that are effectively mitigated using multi-frame approaches. In contrast, in the included images, patients are typically stationary, remaining in their beds; thus, the results indicate that processing multiple frames do not notably enhance the people counting performance for the patient category.

### 3.4. Effect of Neigbouring Frame Size

**Figure 7:**
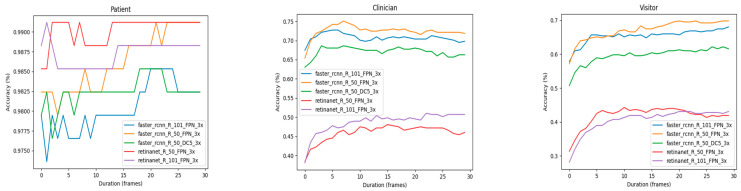
Effect of number of images in multi-frame scenario for Faster R-CNN-Resnet50-FPN architecture. Compared to single-frame analysis, multi-frame analysis tends to enhance accuracy, with categories involving movement, such as visitors and clinicians, being more significantly affected by the number of frames used.

Figure 7 shows the effect of the length of frame intervals on counting performance. In this experiment, the people counting was conducted 30 times utilizing different intervals. It is apparent that increasing the frame interval can mitigate inaccuracies caused by occlusions or false alarms, thus improving overall performance. However, the optimal interval for extension differs among object categories. Specifically, extending the interval to 30 frames generally resulted in more accurate patient counts. In contrast, for the clinician and visitor categories, extending beyond 15 frames did not yield significant performance gains. The Faster RCNN variant exceled in counting clinicians and visitors, while Retinanet models performed best in counting patients.

### 3.5. Importance of Pretrained Model

Next, we explore the role of transfer learning techniques in improving the effectiveness of our machine learning model. Specifically, we utilized a pretrained model from the COCO dataset as the base architecture for our training process. This initial model is referred to as the “pretrained model.” To assess the impact of this pretraining, we conducted a comparative analysis with a control set of experiments termed “No-pretraining,” where the model training started with randomly initialized weights. The results, detailed in Table 4, illustrate a noticeable performance gap between models trained with and without pretraining. Pretraining markedly improved the capabilities of our model, particularly in the realms of object detection and people counting. For instance, in object detection tasks, the introduction of pretraining led to an average improvement of 10 percent in detection accuracy. The benefits of pretraining are even more significant in people counting, especially in scenarios involving single-frame analysis, as opposed to multi-frame or tracking-based counting methods. These results underscore the critical role of pretraining in improving the performance of models tasked with object detection and people counting.

### 3.6. Preliminary Exploration of Relationship between Delirium and People Counting

**Figure 8:**
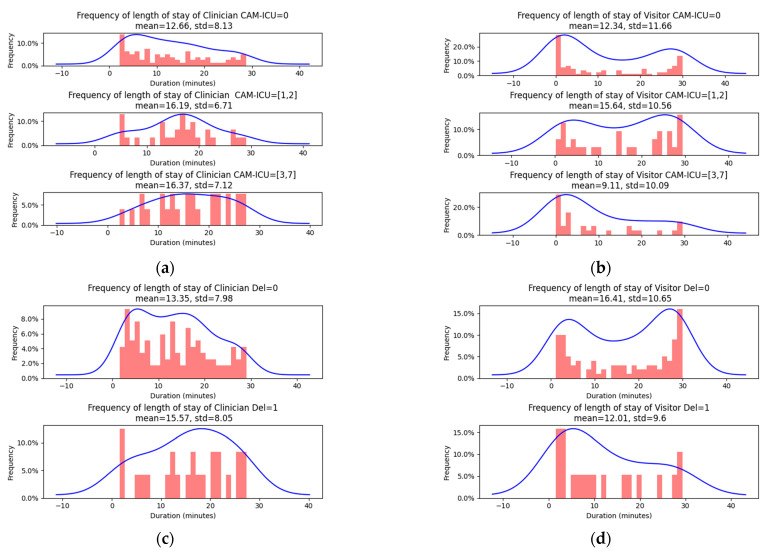
Potential relationship with delirium. (**a**) displays the duration of the clinician in the room by the delirium severity level (0–7), (**c**) displays the duration of the clinician presence between CAM-ICU negative and positive assessments. (**b**,**d**) display the same measurements for the visitor category. The analysis includes a total of **154** assessments, involving **125** non-delirious patients and **29** delirious patients.

In this section, we explore the relationship between the spatial distribution of people within an ICU setting and the occurrence of delirium among patients. We utilize the sophisticated algorithm designed for tracking and counting individuals, applied specifically during a 30 min window centered on each of the 154 delirium assessments conducted, involving 125 non-delirious assessments and 29 delirious assessments. To maintain high performance, our algorithm divided each 30 min interval into 5 s segments, calculating the average presence for each category of individuals—visitors and clinicians—during these segments. The accumulated data from all 360 segments provide a comprehensive overview of the density and movement patterns of people in the ICU. As depicted in Figure 8 the plots reveal a potential link between the presence of people and delirium. For clinicians, the average length of stay in the ICU room for patients without delirium (CAM-ICU-7 score of 0) was 12.66 min, which increased to 16.37 min for patients with a CAM-ICU-7 score of 3–7, indicating mild-to-severe delirium. This trend was continued when examining the average length of stay of clinicians between positive CAM-ICU assessments and negative CAM-ICU assessments (15.57 versus 13.55 min).

Figure 8a presents an analysis of the duration clinicians spend in a patient’s room, segmented by delirium severity levels, which range from 0 to 7. It was observed that for patients with a CAM-ICU score between 3 and 7, clinicians spent more time on average compared to patients with lower delirium severity. Figure 8b shows an analysis of the correlation between CAM-ICU scores and visitor presence reveals an inverse relationship between the severity of delirium and the time visitors spend with the patient. Specifically, patients exhibiting the most severe delirium, with CAM-ICU scores ranging from 3 to 7, tend to have the least amount of time spent by visitors at their bedside. Figure 8c presents a comparison of the duration of clinician presence between delirious (Del = 1) and non-delirious (Del = 0) patients. The findings indicate that the average length of stay of clinicians for delirious patients was notably higher than for non-delirious patients. Figure 8d provides a comparative analysis of the duration of visitor presence between patients with delirium (Del = 1) and those without delirium (Del = 0). The findings indicate that visitors tend to spend significantly less time with patients experiencing delirium compared to non-delirious patients.

Interestingly, the data indicate a potential inverse relationship with visitors. Figure 8b,d depict that visitors spent an average of 16.51 min in the rooms of non-delirious patients, but only 12.01 min with delirious patients. This contrasting behavior underscores varying interaction patterns, which may be influenced by levels of comfort or ability to interact with the patient in the presence of delirium.

## 4. Discussion

Current Electronic Health Records (EHRs) in hospitals often lack comprehensive data on the environmental conditions surrounding patients, particularly in ICU settings. To bridge this information gap, we aimed to develop a model that could automatically recognize the distribution of people in ICU rooms. In this study, we introduced a straightforward pipeline that employs an object detection model to count people within the room. We also developed three different methods to address the challenges of occlusion in the dynamic nature of ICU environments. Our findings indicate that while larger models like Retinanet-Resnet101-FPN may offer superior performance in detecting individuals, leveraging inference over a series of frames can mitigate these advantages. Consequently, smaller models such as Faster R-CNN-Resnet50-FPN have shown to deliver satisfactory performance in people counting tasks.

The effectiveness of our people counting approach is closely tied to the duration of the frames used for analysis. Our results indicate that using a duration of 15 frames yields reasonably accurate counts for both visitor and clinician categories. This finding highlights the importance of selecting an appropriate frame length to balance between processing time and counting accuracy. In comparison to the multi-frame approach, object tracking showed superior performance in counting accuracy. This improvement is largely due to the method’s ability to maintain the identity of individuals across different frames, thereby enhancing the continuity and reliability of the count. Consistent with previous studies [23,30], our study also demonstrates the benefits of adopting transfer learning strategies. By leveraging a pretrained model, we were able to significantly enhance the accuracy of our detection model. This increase in detection accuracy naturally extended to improvements in people counting performance, as more accurate detection facilitates more reliable and precise counting.

Due to an insufficient sample size, we were not able to fully examine the relationship between delirium presence (yes/no), delirium severity, and model output. Our findings align with previous studies in that report clinicians spend more time in the rooms of patients with delirium, compared to patients without delirium [6]. The relationship between the visitor duration and the presence of delirium requires further investigation. If substantiated in future research studies, it is a concerning finding as family presence has shown to significantly decrease the risk of delirium [31]. In the future, if the algorithm identifies a low visitor count, a recommendation provided to clinicians could be to involve family.

While ambient sensor data and intelligent algorithms have potential to inform clinical care, it is critical to evaluate the privacy and ethical concerns of such algorithms. The end-user of this algorithm may be a clinician or an administrator using forecasting to allocate resources to specific patients. Future studies should define a use case and explore the ethical and privacy considerations for such an algorithm to ensure that the benefits outweigh any risks to patients or clinicians. Many recent publications outline frameworks for such an evaluation [32,33].

This study is not without limitations. Our dataset predominantly featured images from ICUs with only one patient per room, which may not reflect more complex scenarios. Additionally, the objects in our images were annotated using bounding boxes rather than pixel-wise segmentation. Although bounding box annotations allow for quicker data processing given our limited number of annotators, they might not be as precise as pixel-wise annotations. However, for the purpose of people detection in our context, pixel-wise tracking does not significantly enhance model performance. Another challenge we faced was inconsistencies in the timestamps between the video recordings and patient delirium assessments due to cameras not being properly synchronized or an internal camera issue. To ensure data integrity, we excluded any assessments where the discrepancy between the recording time and the assessment exceeded 30 min. As a result, our analysis included a total of 154 assessments, comprising 125 non-delirious and 29 delirious patients. This misalignment also affected the volume of usable data; for instance, only 29 assessments involving delirious patients and 125 involving non-delirious patients were included. The limited data volume restricted the depth and robustness of our findings. A larger dataset of assessments is needed to formally evaluate statistical relationships and better understand the interplay between patient conditions and environmental factors.

Our method was specifically implemented for people counting in ICU rooms, an area where, to the best of our knowledge, no similar work currently exists. Despite the lack of direct comparisons, we can relate individual components of our approach to existing research in related domains such as people counting via detection, density estimation, and regression methods. Density estimation techniques typically utilize an L2 norm loss function to train deep learning models by minimizing the error between the predicted density map and the ground truth, often employing network architectures such as VGG, Inception, and Resnet. Regression-based approaches generally involve three main steps: extracting the foreground, deriving features from the foreground—such as crowd masks, edge counts, and texture features—and applying regression models such as linear or piece-wise linear functions, ridge regression models, or neural networks to estimate crowd counts. In contrast, our method treats people counting as a simple detection problem, which we believe is more suitable for ICU environments. This strategy not only enhances the transparency of the algorithm but also improves computational efficiency. Our object detector first identifies people across different categories and then counts them using single-frame or multi-frame analysis and tracking mechanisms, providing more effective solutions for the unique challenges presented by ICU settings. By performing people counting through object detection, we gain several benefits. First, our pipeline becomes architecture-independent, allowing us to use any detection model for people counting. For instance, in this work, we utilized the Retinanet detection model, which incorporates focal loss as part of its training mechanism. Additionally, integrating a detection model into our pipeline enables the generation of detection outputs that are also suitable for surveillance purposes in ICU environments.

Follow-up studies could enhance the accuracy of tracking people distribution in the ICU. For instance, rather than using a simple Kalman Filter to estimate each person’s position, a sequential preprocessing model such as a Transformer or extended Kalman Filter could account for the correlation between people distribution and the patient’s illness severity.

## 5. Conclusions

The ambient monitoring of ICU rooms presents a valuable opportunity to enhance clinical care by offering real-time insights into the current and anticipated acute needs of patients. This study aimed to develop an artificial intelligence model utilizing computer vision to detect and count the number of individuals (patients, clinicians, and visitors) in an ICU room. To this end, we carefully assembled a dataset by annotating images from intensive care units (ICUs). The dataset encompasses detailed annotations of images taken in the ICU settings for a total of 52 patients. To count the number of people in the room, we employed a counting-by-detection approach using images captured in ICU patient rooms, developing three methods: single-frame, multi-frame, and tracking to count. Our approach was tested on image data from an ongoing prospective study, revealing that the tracking-to-count method provides the most accurate performance for people counting in ICU rooms.

Furthermore, we analyzed the distribution of people in the room at 30 min intervals and descriptively explored these counts with delirium. The findings indicate a potential relationship between the presence of clinicians and the occurrence of delirium, while the duration of visitors’ stay showed a potential inverse relationship, potentially due to visitor comfort. Future studies can further explore these relationships. The use of time-series models may provide insight into how the fluctuations and changes to the number and type of people in the room relate with the onset or progression of delirium. The outcome of this system could enhance patient care protocols based on real-time data processed by our model to improve patient care by aligning staff workflows and interactions with patient needs.

## Figures and Tables

**Table 1:** Model configuration.

Name	Value
Training iterations	16 K
Training batch size	4 images
Training base learning rate	$2.5\times 10^{-4}$
Training size	1704 images
Testing size	426 images

**Table 2:** Object detection performance of different model architectures.

Name	Train Time per Iter.	Inference Time per Iter.	Train Memory	AP	bbox AP (Patient)	bbox AP (Clinician)	bbox Ap (Visitor)
	(Seconds) ^1^	(Seconds) ^1^	(GB) ^1^	(%)			
Faster R-CNN-Resnet50-FPN	0.209	0.038	3.0	52.37	47.95	59.23	49.93
Faster R-CNN-Resnet50-DC5	0.378	0.070	5.0	53.25	48.16	58.78	52.83
Faster R-CNN-Resnet101-FPN	0.205	0.041	4.1	54.06	49.51	59.91	52.77
Retinanet-Resnet50-FPN	0.286	0.051	4.1	54.52	49.40	59.60	54.19
Retinanet-Resnet101-FPN	0.291	0.054	5.2	54.60	51.06	59.30	53.52

^1^ Reported by Detectron2. Abbreviations: AP: average precision (%), iter. = iterations.

**Table 3:** Comparative accuracy and performance metrics of various models.

Method	Object Detection Model	Patient	Accuracy Clinician	Visitor	Patient	MSE Clinician	Visitor	Patient	RMSE Clinician	Visitor	Patient	MAE Clinician	Visitor
	Unit	(%)			Error Rate ∈[0,1]								
Single-frame	Faster R-CNN-Resnet50-FPN	97.65	76.83	70.67	0.023	0.554	0.551	0.153	0.744	0.742	0.023	0.307	0.363
	Faster R-CNN-Resnet50-DC5	97.06	70.96	66.56	0.029	0.563	0.815	0.171	0.750	0.902	0.029	0.369	0.457
	Faster R-CNN-Resnet101-FPN	97.94	75.07	68.03	0.020	0.551	0.571	0.143	0.742	0.756	0.020	0.316	0.390
	Retinanet-Resnet50-FPN	96.18	73.90	65.98	0.038	0.560	0.636	0.195	0.748	0.797	0.038	0.348	0.425
	Retinanet-Resnet101-FPN	97.06	72.43	63.63	0.029	0.527	0.909	0.171	0.726	0.953	0.029	0.340	0.504
Multi-frame ^1^	Faster R-CNN-Resnet50-FPN	97.94	78.29	75.07	0.020	0.328	0.410	0.143	0.573	0.640	0.020	0.240	0.0293
	Faster R-CNN-Resnet50-DC5	97.36	76.24	72.72	0.026	0.363	0.604	0.162	0.603	0.777	0.026	0.269	0.357
	Faster R-CNN-Resnet101-FPN	97.65	76.53	74.19	0.023	0.407	0.384	0.151	0.638	0.619	0.023	0.278	0.296
	Retinanet-Resnet50-FPN	97.65	77.12	75.36	0.023	0.434	0.434	0.153	0.658	0.658	0.023	0.287	0.299
	Retinanet-Resnet101-FPN	97.06	77.41	71.55	0.029	0.346	0.554	0.171	0.588	0.744	0.029	0.258	0.354
Tracking to count ^2^	Faster R-CNN-Resnet50-FPN	97.06	75.95	78.59	0.029	0.348	0.290	0.171	0.590	0.538	0.029	0.272	0.237
	Faster R-CNN-Resnet50-DC5	97.36	76.83	77.71	0.026	0.337	0.331	0.162	0.580	0.575	0.026	0.260	0.255
	Faster R-CNN-Resnet101-FPN	95.01	77.71	78.00	0.049	0.334	0.299	0.223	0.578	0.546	0.049	0.258	0.246
	Retinanet-Resnet50-FPN	96.48	77.41	79.17	0.035	0.275	0.225	0.187	0.525	0.475	0.035	0.240	0.214
	Retinanet-Resnet101-FPN	96.77	76.83	77.41	0.032	0.284	0.260	0.179	0.533	0.510	0.032	0.249	0.237

^1^ Duration = 5 frames and Threshold = 0.3. ^2^ Duration = 5 frames and Threshold = 0.3.

**Table 4:** Importance of transfer learning on Faster RCNN Resnet50 FPN.

Object Detection Performance (AP, %)			People Counting Performance (Accuracy, %)			
	Pretrained	No Pretraining	Method/Category		Pretrained	No Pretraining
AP	52.37	44.26	Single-frame	Patient	97.65	96.77
				Clinician	76.53	67.74
Bbox AP (Patient)	47.95	41.47		Visitor	72.72	60.41
			Multi-frame	Patient	97.06	97.65
Bbox AP (Clinician)	59.23	49.59		Clinician	72.43	71.84
				Visitor	78.59	72.43
			Tracking to count	Patient	97.06	98.53
Bbox AP (Visitor)	49.95	41.72		Clinician	75.95	73.31
				Visitor	78.59	75.95

## Data Availability

Data may be available upon request to the corresponding author, Dr. Heidi Lindroth: lindroth.heidi@mayo.edu.
